# A Case for Revisiting the Safety of Pesticides: A Closer Look at Neurodevelopment

**DOI:** 10.1289/ehp.7940

**Published:** 2005-09-07

**Authors:** Theo Colborn

**Affiliations:** 1 University of Florida, Gainesville, Florida, USA; 2 TEDX (The Endocrine Disruption Exchange) Inc., Paonia, Colorado, USA

**Keywords:** adverse effects, behavior, chlorpyrifos, fetal development, human function, neurodevelopment, pesticides, toxicity

## Abstract

The quality and quantity of the data about the risk posed to humans by individual pesticides vary considerably. Unlike obvious birth defects, most developmental effects cannot be seen at birth or even later in life. Instead, brain and nervous system disturbances are expressed in terms of how an individual behaves and functions, which can vary considerably from birth through adulthood. In this article I challenge the protective value of current pesticide risk assessment strategies in light of the vast numbers of pesticides on the market and the vast number of possible target tissues and end points that often differ depending upon timing of exposure. Using the insecticide chlorpyrifos as a model, I reinforce the need for a new approach to determine the safety of all pesticide classes. Because of the uncertainty that will continue to exist about the safety of pesticides, it is apparent that a new regulatory approach to protect human health is needed.

The U.S. Environmental Protection Agency’s (EPA) Office of Pesticide Programs (OPP) estimated that 891 pesticide active ingredients were registered in 1997 (Aspelin and Grube 1999) and that 888 million pounds of pesticide active ingredients were used in the United States in 2001 (Kiely et al. 2004). Few of these chemicals are applied alone but rather are applied in formulations using different combinations of several pesticide active ingredients (MeisterPRO 2004). It is not uncommon for many classes of pesticides, such as insecticides, herbicides, and fungicides, to be used on the same crop (National Agricultural Statistics Service 2005). In the case of insecticides, an adjuvant is often added to the formulations to enhance the intensity of the lethal effect. In the case of herbicides, due to the increasing incidence of plant tolerance to a specific pesticide, some formulations now have as many as three active ingredients (MeisterPRO 2004). Each active ingredient has a specific mode of action for controlling a pest, and each active ingredient has its own possible side effects on the wildlife and humans exposed to it. It is impossible to determine the cumulative risk posed to wildlife and humans as the result of releasing vast amounts of pesticide mixtures into the environment.

The quality and quantity of the data about the risk posed to humans by individual pesticides vary considerably. In some instances there are numerous studies about the health effects of a particular pesticide in humans and laboratory animals, and for others there are very few. In general, the longer the active ingredient has been on the market, the greater the number of citations in the peer-reviewed literature. Data are sparse when linking pesticides with neurodevelopmental effects other than for the insecticides chlorpyrifos (CPF), parathion, and 1,1,1-trichloro-2,2-bis(*p*-chlorophenyl)ethane (DDT).

Unlike obvious structural defects, most neurodevelopmental effects cannot be seen at birth or even later in life. Instead, adverse effects on the nervous system are expressed in terms of how an individual behaves or functions. Behavior and function vary considerably from birth through adulthood. Functional deficits are not “on” and “off” conditions but instead range from inconsequential through very mild to very severe to totally debilitating. Consequently, it is difficult to quantify neuro-developmental impairment. Some of the end points used in the laboratory to detect functional impairment of the brain and nervous system are measured at the gene, cell, biochemical, and/or physiologic levels and often require high-tech instrumentation to quantify. At the human level, a battery of tests is continuing to evolve to measure with increasing sensitivity psychomotor, psychologic, clinical, and psychiatric symptoms to better quantify functional impairment.

In this article I have two principal purposes in discussing the inherent risks of using pesticides, the limitations of testing techniques, and the intrinsic incompleteness of all scientific evidence: *a*) to encourage the use of the open literature about the neurodevelopmental effects of all classes of pesticides when setting the criteria for determining their safety and *b*) to encourage a more rigorous regulatory approach to protect human and environmental health in the absence of complete scientific certainty. I begin by presenting unequivocal evidence of pesticide exposure to numerous classes of pesticides during development. This is followed by a section on human epidemiology where only weak data are available linking neuro-developmental impairment with pesticides. Next, I present a case study of how CPF cryptically interferes with brain development one stage after another. This is followed with selected laboratory studies demonstrating that other insecticides as well as other pesticide classes target prenatal brain development similar to CPF and share similar and sometimes diverse impacts on the construction and function of the brain. As the data reveal, not only insecticides but other classes of pesticides, such as herbicides and fungicides, can also interfere with neurodevelopment. In the “Discussion” I challenge the protective value of current pesticide risk assessment strategies in light of the vast numbers of pesticide products on the market with untold numbers of targets and mechanisms of action that can cause neurodevelopmental damage.

## Evidence of Exposure to Pesticides

Improvements in analytical laboratory equipment and testing procedures have made it easier to detect pesticides and their metabolites at very low concentrations in almost all human tissue. From routinely detecting parts per million (milligrams per kilogram) and more recently to as low as parts per trillion (picograms per kilogram), some laboratories are now able to measure concentrations down to parts per quintillion (femtograms per kilogram). The development of noninvasive sampling methods, such as testing for pesticides and their metabolites in urine, has made it possible to monitor pesticide exposure in infants and children. It is fairly safe to say that every child conceived today in the Northern hemisphere is exposed to pesticides from conception throughout gestation and lactation regardless of where it is born. The herbicide 2,4-dichlorophenoxyacetic acid (2,4–D) was found in approximately 50% of semen samples provided by 97 Ontario, Canada, farmers (Arbuckle et al. 1999). The herbicides atrazine, metolachlor, alachlor, and 2,4–D and the insecticides diazinon and the CPF analyte 3,5,6-trichloro-2-pyridinol (TCP) were found in semen of men in central Missouri and in urban Minneapolis, Minnesota (Swan et al. 2003); the insecticides chlordane, dichloro-diphenyldichloroethylene (DDE), heptachlor epoxide, and hexachlorobenzene (HCB) were found in ovarian follicular fluid from women undergoing *in vitro* fertilization in Halifax, Hamilton, and Vancouver, Canada (Jarrell et al. 1993); hexachlorocyclohexane and *p,p*′-DDE were found in amniotic fluid of women undergoing routine amniocentesis in Los Angeles, California (Foster et al. 2000); and nonpersistent pesticides were found in the amniotic fluid of women referred for amniocentesis in the agricultural San Joaquin Valley, California (Bradman et al. 2003). Pesticides were also found in maternal blood, placental, and umbilical cord blood from women experiencing normal births and stillbirths in India (Saxena et al. 1983), from urban and rural mothers during Caesarian section in the Atoya River basin, Nicaragua (Dorea et al. 2001), and from mothers delivering normal and subnormal weight babies (Siddiqui et al. 2003). In addition, pesticides were found in the breast milk of mothers who delivered by Caesarian section in Nicaragua (Dorea et al. 2001), native Alaskan mothers living an indigenous lifestyle (Simonetti et al. 2001), and women living in southwest Greece (Schinas et al. 2000). A median of 8.26 μg/mL CPF (range, 0.40–458.04 μg/mL) was discovered in the meconium of newborns in Manila, Philippines (Ostrea et al. 2002). Six organo-phosphate (OP) pesticide metabolites were found in the meconium of 20 newborns in New York City (Whyatt and Barr 2001). The babies’ first bowel movements held concentrations 10–100 times higher than their cord blood. One metabolite, diethylthiophosphate, was found in all 20 samples; another, diethyl-phosphate, was found in 19 of 20 samples. Both are metabolites of diazinon, CPF, and several other OP insecticides.

An eastern Washington State research team surveyed OP metabolites in the urine of 210 farmworkers and their children and in dust from their homes and vehicles (Coronado et al. 2004). They segregated farm chores into several classes: harvesting and picking, thinning, loading, transplanting, and pruning. Azinphosmethyl, an OP, was more often found in dust in thinners’ homes (92.1% vs. 72.7%) and vehicles (92.6% vs. 76.5%) than in those of workers who did no thinning. Thinners’ children had higher concentrations of OP metabolites in their urine, and the metabolites were found more frequently in the children (91.9% detectable in urine), compared to the adults (81.3% detectable; *p* = 0.002).

In Seattle, Washington, investigators measured five OP metabolites in 24-hr urine samples of preschool children (2–5 years of age) who were raised on either a predominantly organic (*n* = 18) or predominantly conventional diet (*n* = 21) (Curl et al. 2003). Pesticide use was also recorded for each home. Median total dimethylphosphate metabolites (0.06 μmol/L) were significantly higher than median total diethyl alkylphosphates (0.02 μmol/L; *p* = 0.0001) in the urine. Those children on a conventional diet had levels of dimethylphosphate metabolites six times higher than those of children on an organic diet (medians = 0.17 and 0.03 μmol/L, respectively; *p* = 0.0003). Median concentrations of both metabolites were almost an order of magnitude higher in the conventionally fed children (0.34 μmol/L vs. 0.04 μmol/L). There were no age differences in the children in the two groups. Home use of pesticides varied, with seven conventional-diet families using OPs versus three organic-diet families using OPs. Although the study group was small and there were difficulties collecting urine samples, this research provides the first empirical data comparing urinary levels of pesticides in youngsters consuming predominantly organic versus conventional diets.

## Human Epidemiology

Determining a link between fetal exposure to a specific chemical and long-term expression of a change in health poses a monumental challenge when designing epidemiologic studies. For example, one human epidemiologic study uncovered weak but statistically significant associations between neurodevelopmental impairment as a result of exposure to two pesticides during gestation. In a large study of live births (*n* = 1,532), including 536 children fathered by pesticide applicators, Garry et al. (2002) discovered that “adverse neurologic and neurobehavioral developmental effects clustered among the children born to applicators of the fumigant phosphine [odds ratio (OR) = 2.48; 95% confidence interval (CI), 1.2–5.1].” They also discovered an OR for the herbicide glyphosate (Roundup) of 3.6 (95% CI, 1.3–9.6). Among the children in the phosphine group (*n* = 290), two were diagnosed with autism, which is high compared with the prevalence nationwide, and five were diagnosed with attention deficit disorder/attention deficit hyperactivity disorder (ADD/ADHD). It took years of close interaction with the families in this study to be able to track their pesticide exposure without having to resort to recall and to follow the children’s functional development (Garry VF, personal communication). The investigators were cautious about their findings and asked for confirmation.

Another study suggests that CPF might have an effect on head circumference related to the activity of paraoxonase (PON1), an enzyme that can detoxify CPF before it can inhibit acetylcholinesterase (Berkowitz et al. 2004). Babies with a small reduction in head circumference were from mothers whose TCP concentrations were above the detection limit, and their PON1 activity was in the lowest tertile (*p* = 0.014). Mothers and their infants (*n* = 404) were recruited from East Harlem and other sections of New York City.

In a more recent study, Young et al. (2005) looked at the relationship between maternal OP urine metabolites and infant neuro-development. They employed a battery of tests using the Brazelton Neonatal Behavioral Assessment Scale for habituation, orientation, motor performance, range of state, regulation of state, autonomic stability, and reflex in 381 infants younger than 62 days of age. Young et al. (2005) found a significant association between increasing total concentrations of maternal urine OP metabolites representing “approximately 80% of OPs used in the Salinas Valley” and increasing numbers of abnormal reflexes in the infants from days 3 to 62. The median age for testing the infants was day 3. Mothers’ urine was tested at 14 and 26 weeks during gestation and at day 7 post-partum. The median urine levels of dialkyl phosphate (DAP), dimethyl phosphate, and diethyl phosphate, respectively, were 132, 97, and 21 mol/L during gestation and 222, 160, and 27 nmol/L after delivery. DAP represents the total of diethyl and dimethyl phosphate metabolites. The dimethyl metabolites could reflect exposure to malathion, oxydemetonmethyl, dimethoate, naled, and methidathion, and the diethyl metabolites could reflect exposure to diazinon, CPF, and disulfoton used in the Salinas Valley. It is important to keep in mind that the OPs are readily metabolized, and exposure can vary considerably and most often is transient and unpredictable. The authors noted that there were large within-person variations in urine levels in this study.

## A Case Study: The Cryptic Neurodevelopmental Effects of CPF

The insecticide CPF is an OP pesticide that has been on the market since 1965 to control insects in agriculture, gardens, building construction, and households. In 2002 the use of CPF was restricted to only agricultural applications, and all domestic use was to be completely phased out by 1 January 2005. The metabolites of CPF have been widely reported in human tissue. In a study based on data from the Centers for Disease Control and Prevention’s (CDC 2001) first *National Report on Human Exposure to Environmental Chemicals,* Hill et al. (1995) found the CPF analyte TCP in 82% of urine samples (*n* = 1,000) from a broad sample of the U.S. population between the ages of 20 and 59 years from all regions of the country. The CDC’s *Second National Report on Human Exposure to Environmental Chemicals* (CDC 2003) states that the levels of TCP were similar to levels presented in the first *National Report on Human Exposure to Environmental Chemicals* (CDC 2001) but gave no statistics concerning the extent of exposure across the population. Like the other OP insecticides, CPF inhibits the enzyme acetylcholinesterase, which destroys acetylcholine, the neuro-transmitter that activates cholinergic neurons. These are an important group of nerve cells that control signals in the peripheral nervous system and in the brain and spinal cord. If acetylcholine is not inactivated immediately by the activity of acetylcholinesterase, it over-stimulates the neurons, and tremors, convulsions and death can follow.

As scientists probed deeper into the activity of CPF, a wealth of information surfaced from laboratory studies about its effects on the development and function of the brain and nervous system in embryos, fetuses, and young animals. Although many of the studies were performed on rats and there are differences in the ontogeny of specific parts of the brain between rats and humans, the development of the rat brain through postnatal day (PND) 21 provides a model for the development of the human brain through to birth.

A series of reports starting in 1991 confirmed that CPF is a cholinesterase inhibitor and that neonatal rats were more sensitive than adults when exposed to a single maximum tolerated dose (Pope and Chakraborti 1992; Pope et al. 1991, 1992). These studies also confirmed that the fetus recovers quicker than the adult from cholinesterase inhibition, suggesting that the fetus would be protected from CPF if all the adverse effects were due to cholinesterase inhibition alone. Lassiter et al. (1998), however, wrote that although the fetus could recover faster between repeated doses of CPF, this was only an “illusion that the fetal compartment is less affected than the maternal compartment.” Realizing that something other than cholinesterase inhibition was affecting the fetus, a team from Duke University led by Theodore Slotkin gradually began to demonstrate that other mechanisms of action of CPF alter prenatal development of the brain and behavior and that the embryo and fetus are sensitive to cholinesterase inhibition at doses that would not be toxic to an adult (Qiao et al. 2003; Slotkin 2004). These studies provided information about how the brain develops and functions and also provided a chronology of how CPF interferes at successional stages of brain development (Qiao et al. 2002). This team also demonstrated that CPF-oxon, the active metabolite of CPF, is the compound that causes cholinesterase inhibition and that the actual neuroteratogen is CPF (see Slotkin 2004 for a step-by-step description of how their CPF research progressed).

Slotkin and colleagues demonstrated that as the brain and nervous system are constructed and programmed, there are numerous points in time and at sites where CPF could interfere. CPF attacks the neurons that appear in the earliest stage of brain and central nervous system (CNS) development (Qiao et al. 2004). Neurons process information and are the signaling or transmitting elements in the nervous system. Damage to neurons at this early stage may not be expressed until years later. For example, a brief subtoxic dose of CPF [1 or 5 mg/kg body weight (bw)/day] during neurulation can cause behavioral alterations during adolescence and adulthood (Icenogle et al. 2004). And, although some early symptoms of CPF exposure disappear during certain stages of development, different neurologic symptoms can appear later in life (Qiao et al. 2002, 2003, 2004).

Glial cells that appear later than neurons during early development were shown to be more vulnerable than neurons to CPF (Qiao et al. 2002; Roy et al. 2004). There are more than twice as many glial cells (> 200 billion) in the body than neurons. Glial cells come in many varieties; they are supportive cells critical for normal development and function and serve as a “scaffold” for migration of cells during tissue construction [see Barone et al. (2000) on brain development]. Glial cells also provide nutrition to the neurons and provide a link with the immune system, responding to damage by acting as scavengers of pathogens and neuronal debris. CPF preferentially targets the glial cells among the cells it attacks (Garcia et al. 2002).

Slotkin and colleagues repeatedly demonstrated that CPF toxicity is not limited to cholinesterase inhibition alone but can act by other mechanisms. For example, *in vitro* and *in vivo* studies at three levels of development from DNA to the cell and the whole animal revealed that CPF is far more toxic than previously thought because of this wider range of activity (Crumpton et al. 2000). CPF impairs the binding to DNA of nuclear transcription factors (AP-1 and Sp1) that modulate cell replication and differentiation. When undifferentiated and differentiated neurons were exposed to CPF, the response of some transcription factors varied. Although the activity of one set of cells might not be affected, the activity of another set of cells might be significantly reduced. An independent study at Johns Hopkins University (Schuh et al. 2002) confirmed the ability of CPF to alter the activity of another nuclear transcription factor in cortical neurons, the Ca^2+^/cAMP response element binding protein (CREB), which is critical for cell survival and differentiation during development and is critical for memory. CPF increased the activated level of CREB at 0.01 nM, well below the level at which cholinesterase inhibition is expressed and below the typical level of human exposure. Schuh et al. (2002) also demonstrated that CPF-oxon did not cause the alteration, supporting the conclusion of Crumpton et al. (2000) that CPF is more than a cholinesterase inhibitor. Crumpton et al. (2000) also demonstrated that the CPF effects on the development of the forebrain in the rat, which reaches its peak stage of development during gestation, were not as severe as the effects on the cerebellum, which reaches its peak 2 weeks after birth. The cerebellar changes in the later stages of development, however, could not have been the result of cholinesterase inhibition because the cerebellum is not innervated with cholinergic receptors like the forebrain is (Crumpton et al. 2000).

Much of the research undertaken by Slotkin and colleagues demonstrated that models of adult toxicity do not extrapolate to fetuses and would not predict the vulnerability of the embryo to TCP and CPF (Aldridge et al. 2004, 2005a). The ever-changing state of the embryo makes it a more sensitive model for toxicity and a better predictor of long-term, delayed effects. Slotkin and colleagues have demonstrated that the embryo and fetus reveal innumerable mechanisms of action of toxicity that could not be detected in an adult animal. For example, in a series of *in vitro* studies, a 25% increase in reactive oxygen species (ROS) was found 10 min after undifferentiated glial C6 cells were exposed to CPF (Garcia SJ et al. 2001). During some stages of development, selected regions of the brain are vulnerable to CPF by interference with the G-protein in the adenylyl cyclase (AC) cascade by disrupting nuclear transcription DNA binding (Meyer et al. 2003; Slotkin 1999). CPF caused abnormal tissue/cell development in cultured rat embryos through vacuolation of the cytoplasm (Roy et al. 1998). CPF, CPF-oxon, and TCP inhibit DNA synthesis in PC12 cells (typical neuronal cells) and C6 cells (typical glial cells), having a greater effect on the glial cells, with the exception of the TCP (Qiao et al. 2001). Qiao et al. (2001) also showed that CPF is a stronger DNA synthesis inhibitor than CPF-oxon, although it is a weaker cholinesterase inhibitor. Confirming again that certain regions of the developing brain were more susceptible than others, Qiao et al. (2001) found that CPF and TCP suppress DNA synthesis in the epithelium of the forebrain and inhibit neural cell replication. These studies also revealed that serum binding proteins can be protective of DNA antimitotic activity, but because fetuses and newborns have lower concentrations of serum proteins than adults, they could be more vulnerable.

In a series of whole-animal studies looking at damage in rats from the embryo to the adult, Slotkin and colleagues demonstrated again that assays using adult animals cannot predict the long-term delayed effects in the offspring. For example, within hours after 9.5-day-old embryos were exposed to CPF, they showed clear signs of damage that was restricted to the primordial brain (Roy et al. 1998). Upon histologic examination, Roy et al. (1998) found apoptosis and altered mitotic figures, along with gross disruption of the architecture of the developing brain, all in the absence of any gross morphologic defects in the other parts of the embryo. As these animals matured, CPF damage was demonstrable in a wide variety of brain regions. The most vulnerable target was the hippocampus, with the damage expressed both as deficits in nerve activity and as corresponding behavioral abnormalities (Icenogle et al. 2004). Dosing an adult animal similarly would not have provoked these effects of fetal origin.

The complexity of the toxicity of CPF became more apparent as sex-related differences began to appear in *in vivo* assays. The sex-related changes occur when CPF exposure takes place during gestation days (GD) 17–20 (late gestation) and PND1–4 and again at PND11–14. The timing of this exposure in the rat is comparable to human brain development during the perinatal and neonatal period (Aldridge et al. 2004; Meyer et al. 2004a; Slotkin et al. 2001). Late prenatal exposure to CPF has also been shown to cause long-term sex-specific changes in cognitive performance (Levin et al. 2002). Adolescent and adult females were more vulnerable to CPF, based on their number of errors during working- and reference-memory tasks. Levin et al. (2002) also found profound differences between animals exposed to 1 mg/kg and 5 mg/kg CPF, reflecting a U-shaped dose curve. The lowest dose was the most potent in this case, although the highest dose caused the most inhibition of fetal brain cholinesterase. The non-monotonic dose–response curve discovered in the assay, combined with the fact that the results were not dependent on cholinesterase inhibition, raises questions about indirect effects of CPF and its metabolites on the endocrine system via the brain. However, as Slotkin (personal communication) pointed out, hormesis cannot be ruled out until further research proves otherwise. In light of their findings, Levin et al. (2002) noted the need for childhood and adolescent maturation studies and for the development of more sex-selected end points.

At a concentration somewhat higher than human exposure, 50 μg/mL CPF *in vitro* induces the release of norepinephrine from rat brain synaptosomes (Dam et al. 1999). Studies using whole animals confirmed that the release of norepinephrine inhibits synaptogenesis, a condition that persists to adulthood and is sex specific, long after exposure ceases and cholinesterase activity is restored (Levin et al. 2002). Aldridge et al. (2004) showed that CPF administered during GD9–12 up-regulated serotonin (5-hydroxytryptamine; 5-HT) receptors (5-HT-1 and 5-HT-2) and interfered with the 5-HT protein transporter from the neural tube stage through to adulthood. But during GD17–20, CPF initiated larger effects in regions with greater numbers of 5-HT nerve terminals, which were found more in males. This response continued through PND1–4. In contrast, the 5-HT protein transporter was downregulated in females (Aldridge et al. 2004). Aldridge et al. (2005a,b) performed studies demonstrating abnormalities of 5-HT–related behaviors in developing rats exposed to CPF. The research that preceded this report mapped out the ontogeny of serotonin receptors in the brainstem and forebrain (Aldridge et al. 2003). The authors pointed out that serotonin disruption has been linked to appetitive and affective disorders, and the biologic significance of these findings needs to be clarified. These disorders have been the focus of increasing research attention in recent years as the result of the increasing use of prescription and and illicit mind-altering drugs.

## Other Pesticide Products That Interfere with Neurodevelopment

There are numerous opportunities during gestation where insecticides and products from several other chemical classes can alter the purpose of a cell, tissue, organ, or system function in the brain or CNS, much like the discoveries presented for CPF.

### Herbicides.

Over the past 15 years, an Argentinian research team has produced a series of reports on 2,4-D that is comparable to the research on CPF. This team discovered that exposure during lactation to the herbicide 2,4-DBE (the butyl ester of 2,4-D) can alter brain production of 5-HT and its metabolite, 5-hydroxyindoleacetic acid (5-HIAA), in adulthood (Bortolozzi et al. 2001; Evangelista de Duffard et al. 1990; Garcia G et al. 2001). Concentrations of both dopamine and serotonin changed transiently if the animals were exposed only through birth (69 mg/kg bw/day from GD6 to birth; 15 days) and permanently if delivered to the offspring through breastfeeding as well from GD6 to weaning (30 days). Duffard et al. (1996) and Rosso et al. (2000) found that 2,4-D interfered with myelination in the brain as the result of lactational exposure. This caused changes in behavior patterns that included apathy, reduced social interaction, repetitive movements, tremors, and immobility in pups exposed to 2,4-D (Bortolozzi et al. 1999; Evangelista de Duffard et al. 1995). They also discovered that the serotoninergic and dopaminergic effects occurred during postnatal brain development, similar to the effects of CPF. Bortolozzi et al. (1999) and Evangelista de Duffard et al. (1995) also found 2,4-D in breast milk of 2,4-D–fed mothers and in the stomach content, brain, and kidney of 4-day-old pups (Sturtz et al. 2000).

### Insecticides.

Cassidy et al. (1994) reported that the lowest dose of chlordane used in their studies (100, 500, 5,000 ng/g/day both prenatally and postnatally) caused a dose-dependent reduction in testosterone levels in females in adulthood. The lowest dose they used was 10 times lower than the U.S. EPA’s lowest observed adverse effect level (LOAEL) for neurologic effects (1,000 ng/g) and 50 times lower than the U.S. EPA’s LOAEL for developmental effects (5,000 ng/g) of chlordane (Cassidy et al. 1994). Females exhibited improved spatial abilities and auditory startle-evoked responses more similar to male responses, and slight increases in body weight. Changes in male mating behavior included shortening of latency to intromission and increased intromissions. The authors speculated that pesticides structurally similar to chlordane cause masculinization of function and behavior in both sexes because the pesticides mimic the sex steroids or change their plasma levels through other enzyme systems. The two lower doses in this study prompted greater change than the highest dose for auditory startle response, mating behavior, and body weight.

Methoxychlor (MXC), an insecticide whose toxicity depends on its conversion to several metabolites, was considered to be an estrogen for many years and only recently was discovered to have antiestrogenic and androgenic properties as well. To measure neuro-developmental impacts, Palanza et al. (2002) fed pregnant CD-1 mice environmentally relevant doses of MXC (0.02, 0.2, and 2.0 μg/g mother bw/day) from GD11 to GD17 and examined them on postpartum days 2–15. Mothers fed the lowest dose spent less time nursing than the controls, possibly reflecting the inverted U-shaped dose–response curve expressed by endocrine disruptors. At late adolescence the pups exhibited a reduction in novelty seeking (both the environment and objects), with a difference between males and females (Palanza et al. 1999). Male sexual aggression was reduced at puberty but returned to normal in adulthood. The reduction in aggressive behavior in the periadolescent male CD-1 mouse as a result of MXC exposure (20 μg/kg/day) occurred at a dose 100 times lower than the dose at which the Agency for Toxic Substances and Disease Registry (ATSDR 2002) deemed would cause no harm to humans in 1994. The ATSDR recently withdrew this minimum risk level in light of new evidence on MXC.

Dopaminergic neurons in the substantia nigra project to and release dopamine to the corpus striatum of the brain. This section of the brain integrates neuromuscular and behavioral information and is involved in the control of locomotor activity, exploration, and novelty-induced behavior. It also influences social–sexual interactions such as aggression and maternal behavior. The loss of dopamine function in the neurons connecting the corpus striatum with the midbrain of humans is the cause of Parkinson disease. Male offspring of mice exposed to 20 μg/kg/day MXC had fewer dopaminelike receptors in their corpus striatum and were less active than control females (vom Saal et al. 2003). Females exposed to the same concentrations showed a malelike profile in reactivity to novelty. Similar changes in males and females were seen in mice exposed to *o,p*′-DDT in the same study. In an unrelated study, Lamberson et al. (2001) discovered increased locomotor behavior in offspring of Sprague-Dawley rats administered 0.5 mg/kg/day MXC throughout gestation.

Prenatal exposure to aldrin also causes delayed neurologic impairment that extends through to adulthood. Castro et al. (1992) administered 1 mg/kg aldrin subcutaneously to female rats daily from conception to birth and tested their pups on PND1–2 and again on PND90. On PND90, the animals showed loss of locomotor control and behavioral change(s). Aldrin was not measurable in the animals at the time they were tested.

Paraoxon is the oxidized metabolite of parathion and a potent OP cholinesterase inhibitor. Chronic paraoxon exposure (0.1, 0.15, or 0.2 mg/kg subcutaneously) during a stage of rapid cholinergic brain development from PND8 to PND20 in male Wistar rats led to reduced dendritic spine density in the hippocampus without obvious toxic cholinergic signs in any of the animals (Santos et al. 2004). Some animals in the two highest dose groups died in the early days of the study. All doses caused retarded perinatal growth, and brain cholinesterase activity was reduced 60% by PND21.

Johansson et al. (1995) showed that a single exposure to a pesticide before or shortly after birth can sensitize the offspring to low doses of other pesticides later in life, even though there are no immediate changes in the structure and function of the nervous system at the time of exposure. Only as the exposed individual matures do irreversible alterations in structure and function become evident. The researchers exposed mice to one dose of DDT (0.5 mg/kg bw orally) on PND10 and then at 5 months of age exposed them to bioallethrin (0.7 mg/kg bw) (Johansson et al. 1995) or paraoxon (0.7 or 1.4 mg/kg bw) for 7 days (Johansson et al. 1996). When tested 2 months later, at 7 months of age, the offspring exhibited changes in spontaneous behavior and cholinergic muscarinic receptor density in the cerebral cortex, which led to impairment in learning and memory (Eriksson and Talts 2000). Again, the neurodevelopmental damage was not seen immediately, but instead took 2 months to be expressed. PND10 in the mouse is equivalent to the end of the second trimester in the human. It is during this stage, from the third trimester of pregnancy through 2 years of age in humans, when the neurotransmitter system in the CNS goes through a growth spurt (Eriksson 1997). Throughout these studies the animals showed no clinical signs of toxic symptoms, and the doses used for adult treatment in these studies had no immediate effect on the adult. The dose of DDT used in this study is in the range that human infants might be exposed to during lactation today (Smith 1999). Even though the functional and structural outcomes in the above studies are similar, it should be remembered that they were caused by different mechanisms. For example, bioallethrin causes harm by prolonging sodium channel openings, whereas paraoxon inhibits acetylcholinesterase activity; but they both caused similar neuronal changes, which raises questions about the combined effects of pesticide mixtures on development. These studies support the premise that the differences in susceptibility of adults to pesticides may not be genetic, but rather that susceptibility to pesticides can be acquired by low-dose pesticide exposure earlier in life.

### Insecticide and acaricide.

Rat pups displayed deficits in learning and retention of memory after exposure to the organochlorine insecticide and acaricide endosulfan (6 mg/kg bw) on PND2–25 (Lakshmana and Raju 1994). The concentrations of the neurotransmitters, noradrenalin, dopamine, and serotonin in the olfactory bulb, hippocampus, visual cortex, brainstem, and cerebellum either increased or decreased depending on the days of examination, PND10 and PND25. The authors ruled out acetylcholinesterase inhibition as the cause of the alterations in the production of the neurotransmitters because they found no differences in acetylcholine activity in any of the regions of the brain used in the study. They suggested that endosulfan directly led to a “re-altering” of the construction of those parts of the brain. By PND25, as the differentiation and organization of the observed tissues proceeded in the presence of endosulfan, the rats’ performance became significantly compromised.

### Fungicides.

Gray and Ostby (1998) provided an excellent overview of how prenatal exposure to a fungicide can alter sexual behavior and function in adulthood, even though growth and viability are not compromised. The neurobehavioral alterations quantified in the studies they reviewed include activity level, aggression, mounting frequency, and completed intromissions. In a study using the fungicide vinclozolin, Gray et al. (1994) reported that 100% of the exposed males failed to attain intromission, although there was no reduction in mounting behavior. In subsequent studies, newborn male and female rats were injected on PND2 and PND3 with 200 mg/kg vinclozolin and observed for social behavior on PND36 and PND37 (Hotchkiss et al. 2002). Both males and females exhibited changes in play behavior. Females became involved in increased rough-and-tumble play, a behavior imprinted by male hormones in the brain during early development. Conversely, the males’ rough-and-tumble play was reduced, and they behaved more like unexposed females. Because only one dose was used, this study does not indicate the lowest dose needed to initiate these changes. More recently, on PND34 Colbert et al. (2005) found significantly increased nape contact, pounce, pin, and wrestle play behavior in male offspring of females exposed to 6 and 12 mg/kg bw/day vinclozolin from GD14 to PND3. At a maternal dose of 1.5 mg/kg bw/day vinclozolin, there was a significant increase in penile dysfunction in adulthood. Future studies should include more than one dose, preferably over several orders of magnitude, to take into account the susceptibility and sensitivity of the developing animal.

## Discussion

There is a great deal of uncertainty about the neurodevelopmental effects of pesticides among the human studies presented here. Exposure has become too complex because of the hundreds of pesticide active ingredients on the market, confounded by background exposure to industrial chemicals that share similar effects. In addition, functional changes are expressed over a continuum, making it difficult to document the damage which often is expressed as more than one lesion and at different intervals or stages of development. The pesticides discussed here, with the exception of DDT, are still widely used in the United States despite these data. Although this information is available, the U.S. EPA has rarely used the open literature in its risk assessments, generally using only data submitted by manufacturers. Industry continues to use traditional toxicologic protocols that test for cancer, reproductive outcome, mutations, and neurotoxicity, all crude end points in light of what is known today about functional end points. In using manufacturer data, the U.S. EPA misses almost all delayed developmental, morphologic, and functional damage of fetal origin and, in the case of CPF and all OPs, continues to rely primarily on blood cholinesterase inhibition data in risk assessments (Zheng et al. 2000). The U.S. EPA should accept nonguideline, open literature to determine the toxicity of a chemical. For example, Brucker-Davis (1998) published a comprehensive review of the open literature in which she found 63 pesticides that interfere with the thyroid system—a system known for more than a century to control brain development, intelligence, and behavior. Yet, to date, the U.S. EPA has never taken action on a pesticide because of its interference with the thyroid system.

It would be difficult to find another pesticide in use today that has been as systematically studied as CPF. The amazing litany of diverse mechanisms discovered in the series of CPF studies raises serious questions about the safety of not only CPF and the other OPs but all pesticides in use today. Most astounding is the fact that a large part of CPF’s toxicity is not the result of cholinesterase inhibition, but of other newly discovered mechanisms that alter the development and function of a number of regions of the brain and CNS. These findings send a warning that even though an OP pesticide like CPF may have a very high EC_50_ (concentration that produces 50% of the maximum possible effective response) for acute toxicity as a result of cholinesterase inhibition, it may have other toxic strategies that are far more egregious than cholinesterase inhibition. This raises a question about the value of using EC_50_ values if they do not represent the most sensitive end point. Qiao et al. (2003) warn that “developmental neurotoxicity consequent to fetal or childhood CPF exposure may occur in settings in which immediate symptoms of intoxification are absent.” They also point out that in the case of CPF, damage is not always global (referring to the entire brain) but may only interfere in specific regions of the brain during development, which could increase the difficulty of detecting the damage. S.J. Garcia et al. (2001) state that “measurement just of cholinesterase activity is a questionable approach in assigning an appropriate index of safety.”

The knowledge gained from a decade of the CPF/brain studies by Slotkin and colleagues and the 2,4-D/brain studies by Evangelista de Duffard and co-workers not only demonstrates the insidious nature of CPF and 2,4-D exposure, but it also demonstrates the weaknesses in current standard practices for determining the safety of a pesticide or any other synthetic chemical. These discoveries demonstrate that a much larger battery of tests must be used when determining the safety of commercial pesticides. Even a U.S. EPA analysis of developmental neurotoxicity studies stated that the U.S. EPA’s current developmental neurotoxicologic testing protocol is “not a sensitive indicator of toxicity to the offspring” and urged the U.S. EPA “to further consider if it will use literature data” (Makris et al. 1998). In this case, “literature data” refers to all of the peer-reviewed reports concerning the pesticide impacts on neuro-development that heretofore have not been used for risk assessment by the agency. In the case of CPF and 2,4-D, it appears that those who reviewed the data failed to understand its significance or had other reasons to ignore it. The U.S. EPA needs to convene a panel of independent experts to review these studies for applicability to determine if and how they can be used for registration.

Laboratory studies have clearly revealed neurologic damage after exposure to specific pesticides and in some studies at concentrations equivalent to ambient exposure. Even so, the animal testing for regulatory purposes that takes place today does not attempt to detect adverse health effects at the concentrations at which humans are exposed. Instead, the highest concentrations of chemicals tested are those that can be used without killing the animals or reducing the test mother’s weight and her reproductive ability. In most animal studies the pesticides are administered at high oral or subcutaneous doses orally, not reflecting that, for most humans and wildlife, exposure could in many instances be dermal or via inhalation and, in many cases, over a long period of time at low doses. The U.S. EPA currently requires chronic toxicity studies, but it is locked into using high doses to elicit effects and has not overcome the difficulty of detecting effects from chronic or ambient exposure or low doses. In addition, the human pharmacokinetics of pesticide exposure can either enhance or reduce the health impacts depending on individual variations. In some cases the major or minor metabolites are more toxic than the parent compound, which is listed as the active ingredient.

In a recent study, Bowers et al. (2004) found a different profile of developmental neurotoxicity between polychlorinated biphenyls (PCBs; such as Aroclor 1254) alone and with a mixture of organochlorine pesticides. Very low doses of the chemicals together delayed ear opening, affected geotaxis, and reduced grip strength. Ultimately, mortality, growth, thyroid function, and neurobehavioral development were affected. It is safe to say that there are very few people in the developed world today who are not carrying PCBs in their bodies. If animal testing continues to be used for determining the safety of pesticides, at least one group of the test animals should be exposed to PCBs before testing the pesticides for their ability to cause unpredictable interactive effects such as those described above.

It should be pointed out that the same signaling systems (AC cAMP) involved in the sex-selective changes in brain development have also been shown to alter heart and liver function in adulthood (Meyer et al. 2004a, 2004b). The AC system is ubiquitous throughout the body. In the future, the most efficient, comprehensive assays will take advantage of the fact that most chemicals have more than one effect in one system. Cross-disciplinary teams will be required to design these assays so that every organ system is carefully screened for damage. And most important, this will reduce by thousands the numbers of animals needed for testing. However, improved neurodevelopmental tests with laboratory animals will not fulfill their greatest potential if they are not backed up by better batteries of tests to detect functional disabilities in children. Such new, sophisticated quantitative tests are now available and are being updated regularly. These tests go beyond diagnostic testing to “performance evaluation” and are designed to detect the subtle effects of chronic, low-dose exposure (Davidson et al. 2000).

In conclusion, an entirely new approach to determine the safety of pesticides is needed. It is evident that contemporary acute and chronic toxicity studies are not protective of future generations. The range of doses used in future studies must be more realistic, based on levels found in the environment and human tissue. In this new approach, functional neurologic and behavioral end points should have high priority, as well as the results published in the open literature. In every instance, the impacts of transgenerational exposure on all organ systems must be meticulously inventoried through two generations on all contemporary-use pesticides and new pesticide coming on the market. To protect human health, however, a new regulatory approach is also needed that takes into consideration this vast new knowledge about the neurodevelopmental effects of pesticides, not allowing the uncertainty that accompanies scientific research to serve as an impediment to protective actions.

## References

[b1-ehp0114-000010] Aldridge JE, Levin ED, Seidler FJ, Slotkin TA (2005a). Developmental exposure of rats to chlorpyrifos leads to behavioral alterations in adulthood, involving serotonergic mechanisms and resembling animal models of depression. Environ Health Perspect.

[b2-ehp0114-000010] Aldridge JE, Meyer A, Seidler FJ, Slotkin TA (2005b). Alterations in central nervous system serotonergic and dopaminergic synaptic activity in adulthood after prenatal or neonatal chlorpyrifos exposure. Environ Health Perpect.

[b3-ehp0114-000010] Aldridge JE, Seidler FJ, Meyer A, Thillai I, Slotkin TA (2003). Serotonergic systems targeted by developmental exposure to chlorpyrifos: effects during different critical periods. Environ Health Perspect.

[b4-ehp0114-000010] Aldridge JE, Seidler FJ, Slotkin TA (2004). Developmental exposure to chlorpyrifos elicits sex-selective alterations of serotonergic synaptic function in adulthood: critical periods and regional selectivity for effects on the serotonin transporter, receptor subtypes, and cell signaling. Environ Health Perspect.

[b5-ehp0114-000010] Arbuckle TE, Schrader SM, Cole D, Hall JC, Bancej CM, Turner LA (1999). 2,4-Dichlorophenoxyacetic acid residues in semen of Ontario farmers. Reprod Toxicol.

[b6-ehp0114-000010] Aspelin AL Grube AH 1999. Pesticides Industry Sales and Usage: 1996 and 1997 Market Estimates. EPA-733-R-99-001. Washington, DC:Biological and Economics Analysis Division, Office of Pesticide Programs, U.S. Environmental Protection Agency.

[b7-ehp0114-000010] ATSDR 2002. Toxicological Profile for Methoxychlor (Update). Atlanta, GA:Agency for Toxic Substances and Disease Registry. 38252767

[b8-ehp0114-000010] Barone S, Das KP, Lassiter TL, White LD (2000). Vulnerable processes of nervous system development: a review of markers and methods. Neurotoxicology.

[b9-ehp0114-000010] Berkowitz GS, Wetmur JG, Birman-Deych E, Obel J, Lapinski RH, Godbold JH (2004). *In utero* pesticide exposure, maternal paraoxonase activity, and head circumference. Environ Health Perspect.

[b10-ehp0114-000010] Bortolozzi AA, Duffard RO, Evangelista de Duffard AM (1999). Behavioral alterations induced in rats by a pre- and post-natal exposure to 2,4-dichlorophenoxyacetic acid. Neurotoxicol Teratol.

[b11-ehp0114-000010] Bortolozzi A, Evangelista de Duffard AM, Dajas F, Duffard R, Silveira R (2001). Intracerebral administration of 2,4-dichlorophenoxyacetic acid induces behavioral and neurochemical alterations in the rat brain. Neurotoxicology.

[b12-ehp0114-000010] Bowers WJ, Nakai JS, Chu I, Wade MG, Moir D, Yagminas A (2004). Early developmental neurotoxicity of a PCB/organochlorine mixture in rodents after gestational and lactational exposure. Toxicol Sci.

[b13-ehp0114-000010] Bradman A, Barr DB, Henn BGC, Drumheller T, Curry C, Eskenazi B (2003). Measurement of pesticides and other toxicants in amniotic fluid as a potential biomarker of prenatal exposure: a validation study. Environ Health Perspect.

[b14-ehp0114-000010] Brucker-Davis F (1998). Effects of environmental synthetic chemicals on thyroid function. Thyroid.

[b15-ehp0114-000010] Cassidy RA, Vorhees CV, Minnema DJ, Hastings L (1994). The effects of chlordane exposure during pre- and postnatal periods at environmentally relevant levels on sex steroid-mediated behaviors and functions in the rat. Toxicol Appl Pharmacol.

[b16-ehp0114-000010] Castro VL, Bernardi MM, Palermo-Neto J (1992). Evaluation of prenatal aldrin intoxication in rats. Arch Toxicol.

[b17-ehp0114-000010] CDC 2001. National Report on Human Exposure to Environmental Chemicals. Atlanta, GA:Centers for Disease Control and Prevention.

[b18-ehp0114-000010] CDC 2003. Second National Report on Human Exposure to Environmental Chemicals. NCEH Publication no. 02-0716. Atlanta, GA:Centers for Disease Control and Prevention. Available: http://www.cdc.gov/exposurereport [accessed 12 December 2004].

[b19-ehp0114-000010] Colbert NKW, Pelletier NC, Cote JM, Concannon JB, Jurdak NA, Minott SB (2005). Perinatal exposure to low levels of the environmental antiandrogen vinclozolin alters sex-differentiated social play and sexual behaviors in the rat. Environ Health Perspect.

[b20-ehp0114-000010] Coronado GD, Thompson B, Strong L, Griffith WC, Islas I (2004). Agricultural task and exposure to organophosphate pesticides among farmworkers. Environ Health Perspect.

[b21-ehp0114-000010] Crumpton TL, Seidler FJ, Slotkin TA (2000). Developmental neurotoxicity of chlorpyrifos in vivo and in vitro: effects on nuclear transcription factors involved in cell replication and differentiation. Brain Res.

[b22-ehp0114-000010] Curl CL, Fenske RA, Elgethun K (2003). Organophosphorus pesticide exposure of urban and suburban preschool children with organic and conventional diets. Environ Health Perspect.

[b23-ehp0114-000010] Dam K, Seidler FJ, Slotkin TA (1999). Chlorpyrifos releases nor-epinephrine from adult and neonatal rat brain synaptosomes. Brain Res Dev Brain Res.

[b24-ehp0114-000010] Davidson PW, Weiss B, Myers GJ, Cory-Slechta DA, Brockel BJ, Young EC (2000). Evaluation of techniques for assessing neurobehavioral development in children. Neurotoxicology.

[b25-ehp0114-000010] Dorea JG, Cruz-Granja AC, Lacayo-Romero ML, Cuadra-Leal J (2001). Perinatal metabolism of dichlorodiphenyldichloro-ethylene in Nicaraguan mothers. Environ Res.

[b26-ehp0114-000010] Duffard R, Garcia G, Rosso S, Bortolozzi A, Madariaga M, Di Paolo O (1996). Central nervous system myelin deficit in rats exposed to 2,4-dichlorophenoxyacteic acid throughout lactation. Neurotoxicol Teratol.

[b27-ehp0114-000010] Eriksson P (1997). Developmental neurotoxicity of environmental agents in the neonate. Neurotoxicology.

[b28-ehp0114-000010] Eriksson P, Talts U (2000). Neonatal exposure to neurotoxic pesticides increases adult susceptibility: a review of current findings. Neurotoxicology.

[b29-ehp0114-000010] Evangelista de Duffard AM, Bortolozzi A, Duffard RO (1995). Altered behavioral responses in 2,4-dichlorophenoxyacetic acid treated and amphetamine challenged rats. Neurotoxicology.

[b30-ehp0114-000010] Evangelista de Duffard AM, de Alderete MN, Duffard R (1990). Changes in brain serotonin and 5-hydroxyindolacetic acid levels induced by 2,4-dichlorophenoxyacetic butyl ester. Toxicology.

[b31-ehp0114-000010] Foster W, Chan S, Platt L, Hughes C (2000). Detection of endocrine disrupting chemicals in samples of second trimester human amniotic fluid. J Clin Endocrinol Metab.

[b32-ehp0114-000010] Garcia G, Tagliaferro P, Bortolozzi A, Madariaga MJ, Brusco A, Evangelista de Duffard AM (2001). Morphological study of 5-HT neurons and astroglial cells on brain of adult rats perinatal or chronically exposed to 2,4-dichlorophenoxy acetic acid. Neurotoxicology.

[b33-ehp0114-000010] Garcia SJ, Seidler FJ, Crumpton TL, Slotkin TA (2001). Does the developmental neurotoxicity of chlorpyrifos involve glial targets? Macromolecule synthesis, adenylyl cyclase signaling, nuclear transcription factors, and formation of reactive oxygen in C6 glioma cells. Brain Res.

[b34-ehp0114-000010] Garcia SJ, Seidler FJ, Qiao D, Slotkin TA (2002). Chlorpyrifos targets developing glia: effects on glial fibrillary acidic protein. Brain Res Dev Brain Res.

[b35-ehp0114-000010] Garry VF, Harkins ME, Erickson LL, Long-Simpson LK, Holland SE, Burroughs BL (2002). Birth defects, season of conception, and sex of children born to pesticide applicators living in the Red River Valley of Minnesota, USA. Environ Health Perspect.

[b36-ehp0114-000010] Gray LE, Ostby J (1998). Effects of pesticides and toxic substances on behavioral and morphological reproductive development: endocrine versus nonendocrine mechanisms. Toxicol Ind Health.

[b37-ehp0114-000010] Gray LE, Ostby JS, Kelce WR (1994). Developmental effects of an environmental antiandrogen—the fungicide vinclozolin alters sex differentiation of the male rat. Toxicol Appl Pharmacol.

[b38-ehp0114-000010] Hill RH, Head SL, Baker S, Gregg M, Shealy DB, Bailey SL (1995). Pesticide residues in urine of adults living in the United States: reference range concentrations. Environ Res.

[b39-ehp0114-000010] Hotchkiss AK, Ostby JS, Vandenbergh JG, Gray LE (2002). Androgens and environmental antiandrogens affect reproductive development and play behavior in the Sprague-Dawley rat. Environ Health Perspect.

[b40-ehp0114-000010] Icenogle LM, Christopher NC, Blackwelder WP, Caldwell DP, Qiao D, Seidler FJ (2004). Behavioral alterations in adolescent and adult rats caused by a brief subtoxic exposure to chlorpyrifos during neurulation. Neurotoxicol Teratol.

[b41-ehp0114-000010] Jarrell JF, Villeneuve D, Franklin C, Bartlett S, Wrixon W, Kohut J (1993). Contamination of human ovariam follicular fluid and serum by chlorinated organic compounds in three Canadian cities. Can Med Assoc J.

[b42-ehp0114-000010] Johansson U, Fredriksson A, Eriksson P (1995). Bioallethrin causes permanant changes in behavioral and muscarinic acetylcholine receptor variables in adult mice exposed neonatally to DDT. Eur J Pharmacol Environ Toxicol Pharmacol.

[b43-ehp0114-000010] Johansson U, Fredriksson A, Eriksson P (1996). Low-dose effects of paraoxon in adult mice exposed neonatally to DDT: changes in behavioural and cholinergic receptor variables. Environ Toxicol Pharmacol.

[b44-ehp0114-000010] Kiely T Donaldson D Grube A 2004. Pesticides Industry Sales and Usage: 2000 and 2001 Market Estimates. EPA-733-R-04-001. Washington, DC:Office of Pesticide Programs, U.S. Environmental Protection Agency.

[b45-ehp0114-000010] Lakshmana MK, Raju TR (1994). Endosulfan induces small but significant changes in the levels of noradrenaline, dopamine and serotonin in the developing rat brain and deficits in the operant learning performance. Toxicology.

[b46-ehp0114-000010] Lamberson CK, Shavlik LJ, Scalzitti JM (2001). Gestational eco-estrogen administration alters serotonin-2A, D1 and D2 dopamine receptor-mediated behaviors in pups. Soc Neurosci Abstr.

[b47-ehp0114-000010] Lassiter TL, Padilla S, Mortensen SR, Chanda SM, Moser VC, Barone S (1998). Gestational exposure to chlorpyrifos: apparent protection of the fetus?. Toxicol Appl Pharmacol.

[b48-ehp0114-000010] Levin ED, Addy N, Baruah A, Elias A, Christopher NC, Seidler FJ (2002). Prenatal chlorpyrifos exposure in rats causes persistent behavioral alterations. Neurotoxicol Teratol.

[b49-ehp0114-000010] Makris S Raffaele K Sette W Seed J 1998. A retrospective analysis of twelve developmental neurotoxicity studies submitted to the US EPA Office of Prevention, Pesticides, and Toxic Substances. Washington, DC:U.S. Environmental Protection Agency.

[b50-ehp0114-000010] MeisterPRO 2004. Crop Protection Handbook. Willoughby, OH:Meister Publishing Co.

[b51-ehp0114-000010] Meyer A, Seidler FJ, Aldridge JE, Tate CA, Cousins MM, Slotkin TA (2004a). Critical periods for chlorpyrifos-induced developmental neurotoxicity: alterations in adenylyl cyclase signaling in adult rat brain regions after gestational or neonatal exposure. Environ Health Perspect.

[b52-ehp0114-000010] Meyer A, Seidler FJ, Cousins MM, Slotkin TA (2003). Developmental neurotoxicity elicited by gestational exposure to chlorpyrifos: When is adenylyl cyclase a target?. Environ Health Perspect.

[b53-ehp0114-000010] Meyer A, Seidler FJ, Slotkin TA (2004b). Developmental effects of chlorpyrifos extend beyond neurotoxicity: critical periods for immediate and delayed-onset effects on cardiac and hepatic cell signaling. Environ Health Perspect.

[b54-ehp0114-000010] National Agricultural Statistics Service 2005. NASS Pesticide Use Data. Available: http://old.ipmcenters.org/data-sources/nass/ [accessed 8 July 2005].

[b55-ehp0114-000010] Ostrea EM, Morales V, Ngoumgna E, Prescilla R, Tan E, Hernandez E (2002). Prevalence of fetal exposure to environmental toxins as determined by meconium analysis. Neurotoxicology.

[b56-ehp0114-000010] Palanza P, Morellini F, Parmigiani S, vom Saal FS (1999). Prenatal exposure to endocrine disrupting chemicals: effects on behavioral development. Neurosci Biobehav Rev.

[b57-ehp0114-000010] Palanza P, Morellini F, Parmigiani S, vom Saal FS (2002). Ethological methods to study the effects of maternal exposure to estrogenic endocrine disrupters—a study with methoxychlor. Neurotoxicol Teratol.

[b58-ehp0114-000010] Pope CN, Chakraborti TK (1992). Dose-related inhibition of brain and plasma cholinesterase in neonatal and adult rats following sublethal organophosphate exposures. Toxicology.

[b59-ehp0114-000010] Pope CN, Chakraborti TK, Chapman ML, Farrar JD (1992). Long-term neurochemical and behavioral effects induced by acute chlorpyrifos treatment. Pharmacol Biochem Behav.

[b60-ehp0114-000010] Pope CN, Chakraborti TK, Chapman ML, Farrar JD, Arthun D (1991). Comparison of in vivo cholinesterase inhibition in neonatal and adult rats by three organophosphorothioate insecticides. Toxicology.

[b61-ehp0114-000010] Qiao D, Seidler FJ, Abreu-Villaca Y, Tate CA, Cousins MM, Slotkin TA (2004). Chlorpyrifos exposure during neurulation: cholinergic synaptic dysfunction and cellular alterations in brain regions at adolescence and adulthood. Brain Res Dev Brain Res.

[b62-ehp0114-000010] Qiao D, Seidler FJ, Padilla S, Slotkin TA (2002). Developmental neurotoxicity of chlorpyrifos: what is the vulnerable period?. Environ Health Perspect.

[b63-ehp0114-000010] Qiao D, Seidler FJ, Slotkin TA (2001). Developmental neuro-toxicity of chlorpyrifos modeled *in vitro*: comparative effects of metabolites and other cholinesterase inhibitors on DNA synthesis in PC12 and C6 cells. Environ Health Perspect.

[b64-ehp0114-000010] Qiao D, Seidler FJ, Tate CA, Cousins MM, Slotkin TA (2003). Fetal chlorpyrifos exposure: adverse effects on brain cell development and cholinergic biomarkers emerge postnatally and continue into adolescence and adulthood. Environ Health Perspect.

[b65-ehp0114-000010] Rosso SB, Garcia GB, Madariaga MJ, Evangelista de Duffard AM, Duffard RO (2000). 2,4-Dichlorophenoxyacetic acid in developing rats alters behaviour, myelination and regions brain gangliosides pattern. Neurotoxicology.

[b66-ehp0114-000010] Roy TS, Andrews JE, Seidler FJ, Slotkin TA (1998). Chlorpyrifos elicits mitotic abnormalities and apoptosis in neuro-epithelium of cultured rat embryos. Teratology.

[b67-ehp0114-000010] Roy TS, Seidler FJ, Slotkin TA (2004). Morphologic effects of subtoxic neonatal chlorpyrifos exposure in developing rat brain: regionally selective alterations in neurons and glia. Brain Res Dev Brain Res.

[b68-ehp0114-000010] Santos HR, Cintra WM, Aracava Y, Maciel CM, Castro NG, Albuquerque EX (2004). Spine density and dendritic branching pattern of hippocampal CA1 pyramidal neurons in neonatal rats chronically exposed to the organophosphate paraoxon. Neurotoxicology.

[b69-ehp0114-000010] Saxena MC, Siddiqui MKJ, Agarwal V, Kuuty D (1983). A comparison of organochlorine insecticide contents in specimens of maternal blood, placenta, and umbilical-cord blood from stillborn and live-born cases. J Toxicol Environ Health.

[b70-ehp0114-000010] Schinas V, Leotsinidis M, Alexopoulos A, Tsapanos V, Kondakis XG (2000). Organochlorine pesticide residues in human breast milk from southwest Greece: associations with weekly food consumption patterns of mothers. Arch Environ Health.

[b71-ehp0114-000010] Schuh RA, Lein PJ, Beckles RA, Jett DA (2002). Noncholinesterase mechanisms of chlorpyrifos neurotoxicity: altered phosphorylation of Ca^2+^/cAMP response element binding protein in cultured neurons. Toxicol Appl Pharmacol.

[b72-ehp0114-000010] Siddiqui MKJ, Srivastava S, Srivastava SP, Mehrotra PK, Mathur N, Tandon I (2003). Persistent chlorinated pesticides and intra-uterine foetal growth retardation: a possible association. Int Arch Occup Environ Health.

[b73-ehp0114-000010] Simonetti J, Berner J, Williams K (2001). Effects of *p,p*′-DDE on immature cells in culture at concentrations relevant to the Alaskan environment. Toxicol In Vitro.

[b74-ehp0114-000010] Slotkin TA (1999). Developmental cholinotoxicants: nicotine and chlorpyrifos. Environ Health Perspect.

[b75-ehp0114-000010] Slotkin TA (2004). Guidelines for developmental neurotoxicity and their impact on organophosphate pesticides: a personal view from an academic perspective. Neurotoxicology.

[b76-ehp0114-000010] Slotkin TA, Cousins MM, Tate CA, Seidler FJ (2001). Persistent cholinergic presynaptic deficits after neonatal chlorpyrifos exposure. Brain Res.

[b77-ehp0114-000010] Smith D (1999). Worldwide trends in DDT levels in human breast milk. Int J Epidemiol.

[b78-ehp0114-000010] Sturtz N, Evangelista de Duffard AM, Duffard R (2000). Detection of 2,4-dichlorophenoxyacetic acid (2,4-D) residues in neonates breast-fed by 2,4-D exposed dams. Neurotoxicology.

[b79-ehp0114-000010] Swan SH, Kruse RL, Liu F, Barr DB, Drobnis EZ, Redmon JB (2003). Semen quality in relation to biomarkers of pesticide exposure. Environ Health Perspect.

[b80-ehp0114-000010] vom Saal FS Palanza P Colborn T Parmigiani S 2003. Exposure to very low doses of endocrine disrupting chemicals (EDCs) during fetal life permanently alters brain development and behavior in animals and humans. In: Proceedings of Conference: International Seminar on Nuclear War and Planetary Emergencies, 27th Session, August 2002, Erice, Sicily (Ragaini RC, ed). Singapore:World Scientific Publishers, 293–308.

[b81-ehp0114-000010] Whyatt RM, Barr DB (2001). Measurement of organophosphate metabolites in postpartum meconium as a potential bio-marker of prenatal exposure: a validation study. Environ Health Perspect.

[b82-ehp0114-000010] Young JG, Eskenazi B, Gladstone EA, Bradman A, Pedersen L, Johnson C (2005). Association between in utero organophosphate pesticide exposure and abnormal reflexes in neonates. Neurotoxicology.

[b83-ehp0114-000010] Zheng Q, Olivier K, Won YK, Pope CN (2000). Comparative cholinergic neurotoxicity of oral chlorpyrifos exposures in preweanling and adult rats. Toxicol Sci.

